# Clinical outcomes following intravitreal methotrexate for primary vitreoretinal lymphoma

**DOI:** 10.1186/s40942-021-00346-0

**Published:** 2021-12-04

**Authors:** Casey L. Anthony, J. Clay Bavinger, Jessica G. Shantha, Ghazala D. O’Keefe, William A. Pearce, Alfredo Voloschin, Hans E. Grossniklaus, Steven Yeh

**Affiliations:** 1 Department of Ophthalmology, Emory University School of Medicine, Atlanta, GA USA; 2 Department of Medicine, Hematology and Oncology, Emory University School of Medicine, Atlanta, GA USA; 3 Department of Ophthalmology, Truhlsen Eye Institute, University of Nebraska Medical Center, 3902 Leavenworth St., Omaha, NE 68106 USA

**Keywords:** Primary vitreoretinal lymphoma, Primary intraocular lymphoma, Primary central nervous system lymphoma, Methotrexate, Intravitreal, PCNSL, PVRL, PIOL, Uveitis, Masquerade syndrome

## Abstract

**Purpose:**

To describe the visual acuity and anatomic outcomes of intravitreal methotrexate (MTX) for the treatment of primary vitreoretinal lymphoma (PVRL).

**Methods:**

Single-center retrospective case series of patients with a diagnosis of PVRL treated with intravitreal MTX. Patient records were reviewed for demographic information, ocular exam findings, and treatment regimens including number of MTX injections. Clinical outcomes recorded included visual acuity (VA), time to partial (PR) or complete response (CR), disease-free survival, time to relapse, and any CNS progression.

**Results:**

Ten eyes of 7 patients (4 male, 6 female) were reviewed. The mean age ± standard deviation (SD) was 70 ± 12 years. Five patients had prior or concomitant diagnosis of primary CNS lymphoma with a history of systemic chemotherapy including MTX. Three eyes (30%) exhibited isolated vitreous involvement, four (40%) had subretinal lesions, and three (30%) presented with both vitreous and subretinal disease. Mean initial logMAR VA was 0.38 ± 0.52 (Snellen visual equivalent 20/50), while mean final logMAR VA ± SD was 0.34 ± 0.27 (Snellen visual equivalent 20/40) with a mean follow-up time of 26 months (Range, 3–49 months). Patients received an average of 6 intravitreal MTX injections (Range 1–10) over the course of treatment. Two patients received concomitant systemic chemotherapy. Mean time to either PR or CR was 57 days, and 6 eyes (60%) exhibited regression with no relapse after local treatment. For the 4 eyes that eventually relapsed, the mean time ± SD to first relapse was 193 days ± 155 days, and one eye experienced a second relapse. Two of 3 patients with subretinal disease showed complete regression with extended follow-up of 1 and 4 years following treatment with less than 3 doses of intravitreal MTX. One patient with PVRL developed CNS lymphoma during the study period. VA remained stable overall between the initial treatment visit, 3, 6, and 12-months (*P* > 0.05 for paired comparisons of VA over time).

**Conclusions:**

Intravitreal methotrexate was well-tolerated and led to local disease response in the majority of patients at approximately 2 months after initiation of treatment of intraocular lymphoma. Further studies on the efficacy of intravitreal treatment alone versus combined systemic and intravitreal treatment are warranted.

## Introduction

Primary vitreoretinal lymphoma (PVRL), also termed primary intraocular lymphoma (PIOL) occurs when lymphoma cells, most commonly diffuse large B-cell non-Hodgkin lymphoma, proliferate within the posterior segment of the eye, with tissue invasion that may involve the retina, subretinal space, vitreous and/or optic nerve [1, 2]. Approximately 20% of patients have concurrent central nervous system (CNS) involvement, or primary CNS lymphoma (PCNSL), and 90% of patients PVRL will develop CNS lymphoma within 29 months [3]. While considered a rare disease, the incidence of PCNSL has risen over the past decade, contributing to increasing disease prevalence and importance of recognition and treatment [4].

Vision loss can ensue from the accumulation of atypical lymphocytes beneath the retinal pigment epithelium and local retinal ischemia [2]. However, early PVRL or PIOL  is known as a masquerade syndrome, often presenting with vague complaints and insidious onset that can mimic other conditions such as chronic uveitis [5]. Protean symptoms and signs, delayed referrals, and the need for invasive vitrectomy surgery to establish a diagnosis, may contribute to a delay in the diagnosis of PVRL and treatment.

Treatment has evolved over time and may depend on disease location, presence and extent of CNS involvement and patient tolerability to adverse effects associated with treatment. Protocols utilized by clinicians have varied in the literature. Reported multimodal approaches to treatment include systemic therapy, radiotherapy, intravitreal chemotherapy, and biologics such as rituximab [5, 6]. Systemic methotrexate (MTX) is a mainstay of treatment to prevent PIOL progression to extraocular CNS lymphoma. High-dose methotrexate has greatly improved the prognosis of patients with PCNSL; however, therapy can be associated with neurotoxicity [7]. While intravitreal treatment alone mitigates risk of the toxicity of systemic treatment and has been shown to have similar relapse patterns, progression-free survival, or overall survival for patients, many clinicians still use systemic MTX out of concern for subclinical CNS involvement [4, 8–10]. A recent retrospective, multicenter cohort study suggested that extensive treatment (i.e. defined as combinations of systemic and intrathecal chemotherapy, whole-brain radiotherapy, and peripheral blood stem cell transplantation) was not proven to prevent CNSL and associated with more severe adverse effects than local treatment alone (i.e. ocular radiotherapy or chemotherapy) [11].

Intravitreal therapies for ocular disease have been administered on a scheduled basis or as needed. However, knowledge gaps remain regarding visual outcomes associated with MTX injection protocols after diagnosis, relapse and remission rates, and tolerability. In this series, we describe our tertiary referral experience center experience regarding the disease presentation and treatment response of patients with PVRL who were treated with intravitreal methotrexate.

## Methods

A retrospective cohort analysis was performed for all patients diagnosed with PVRL treated with intravitreal MTX at the Emory Eye Center. Diagnosis was established via diagnostic vitrectomy or via clinical examination of patients with a known history of CNS lymphoma and characteristic clinical lesions. Diagnostic vitrectomy was elected in the majority of cases; however, in select cases, diagnostic vitrectomy and retinal biopsy were deferred if these procedures were considered too high-risk owing to the location of the retinal lesions and risk of vision loss (e.g. subfoveal lesions and/or. patient deferral). Exclusion criteria included a diagnosis of systemic non-Hodgkin’s lymphoma (i.e. secondary vitreoretinal lymphoma). Institutional Review Board approval was obtained from Emory University. Human research was conducted according to the Tenets of the Declaration of Helsinki.

Patient records were reviewed for demographic information, ocular exam findings, disease presentation and course, and treatment regimens including number of methotrexate (MTX) injections. Clinical outcomes recorded included initial and final visual acuity (VA), time to partial (PR) or complete response (CR), disease-free survival, time to relapse, number of relapses, and any non-ocular CNS progression. To assess treatment response, we utilized standardized guidelines on response lymphoma for primary CNS lymphoma [12]. We defined *partial response (PR)* as minor RPE abnormalities or a decrease in vitreous cells or retinal infiltrate/subretinal lesions, *stable* partial response as a partial response for at least two clinic visits, *complete response (CR)* as a normal eye exam with completely regressed lesions for at least two clinic visits, and progressive disease as any new or recurrent abnormalities.

Statistical analysis was performed with Microsoft Excel (Microsoft, Redmond, WA) and Stata (2014 Stata Statistical Software Release 14; StataCorp LP, College Station, TX, USA). Descriptive data including demographic data and ocular findings were summarized as frequencies or means with standard deviations as appropriate. Snellen visual acuities were converted to logMAR values for statistical analysis. Kaplan–Meier survival analyses were plotted for time to relapse over the patient’s follow-up period.

## Results

Ten eyes of 7 patients (4 male, 6 female) were reviewed and summarized in Table 1. The mean age ± standard deviation (SD) was 70 ± 12 years (range, 56–85). Five patients had prior or concomitant diagnosis of primary CNS lymphoma with a history of systemic chemotherapy including MTX. Three patients (42%) had bilateral disease (i.e., either bilateral presentation or asynchronous, bilateral disease noted during follow-up). Three eyes (30%) exhibited primarily vitreous involvement at presentation, characterized by vitreous haze and cells. Four (40%) had subretinal lesions and RPE changes on OCT, and three (30%) presented with both vitreous and subretinal disease. Mean initial logMAR VA was 0.38 ± 0.52 (Snellen visual equivalent 20/50), while mean final logMAR VA was 0.34 ± 0.27 (Snellen visual equivalent 20/40) with a mean follow-up time of 26 months (range, 3–49 months). VA remained stable overall between initial treatment and 1, 3, 6, and 12-month follow-up (*P* > 0.05 for paired comparisons); however, the largest VA improvement occurred at the 3-month follow-up, with an average of an approximately 2-line improvement to 20/30.

**Table 1:** Demographics, clinical features, treatments and outcomes

Patient	Age/sex	Affected eye	CNS disease	Systemic treatment	PVRL location	Initial VA	Final VA	Total # MTX	Time to response (months)	Time to relapse (months)	PVRL status at final FU	Total FU (months)
1	57/F	OD	Frontal lobe DLBCL s/p resection	MTX, RIT	VIT	20/20	20/50	4	0.25	–	CR	33
		OS			VIT/SR	20/30	20/70	9**	0.25	14	CR	33
2	72/F	OS	CNS DLBCL	MTX	VIT/SR	20/1000	20/40	8	2.0	–	PR	20
3	69/F	OS	R Parietal DLBCL	MTX	VIT	20/60	20/30	5*	1.6	5	CR	19
4	66/F	OS	R cerebellar DLBCL	MTX, RIT, TMZ	SR	20/60	20/40	1	1.2	–	CR	14
5	58/F	OD	Frontal lobe DLBCL	MTX	VIT	20/25	20/20	3	2.8	–	CR	3
		OS			SR	20/20	20/20	3	4.2	–	CR	4
6	86/F	OS	None	None	SR	20/20	20/40	2	2.8	–	CR	49
7	84/M	OD	DLBCL	MTX	SR	20/70	20/150	10*	2.6	2.6	PR	44
		OS			VIT/SR†,‡	20/30	20/50	6*	1.1	3.9	CR	44

*PVRL* Primary vitreoretinal lymphoma, *VA* Visual acuity, *F* Female, *M* Male, *OD* Right eye, *OS* Left eye, *DLBCL* Diffuse large B-cell lymphoma, *MTX* Methotrexate, *RIT* Rituximab, *TMZ* Temozolamide, *VIT* Vitreous, *SR* Subretinal/Retinal pigment epithelial disease, *FU* Follow-up, *CR* Complete response, *PR* Partial response

^†^Patient 7 presented initially with vitreous involvement OS, and developed subretinal/RPE disease at the time of relapse

^‡^Patient 7 also had a history of wet age-related macular degeneration requiring multiple bevacizumab and aflibercept injections during the disease course

^*^2 total cycles of intravitreal MTX due to relapse, **3 cycles of intravitreal MTX due to 2 relapses OS

Eyes received an average of 5.1 ± 3.1 intravitreal MTX injections (range, 1–10) over the course of treatment. Two patients were also receiving systemic chemotherapy for lymphoma at during their course of intravitreal therapy. The typical interval and duration of injections varied among patients and was based on clinician judgement of disease activity at the exam. Patients typically received injections at monthly intervals until disease regression was achieved. Only one patient (Patient 3) received maintenance injections each month until the patient elected to stop therapy.

**Fig. 1:**
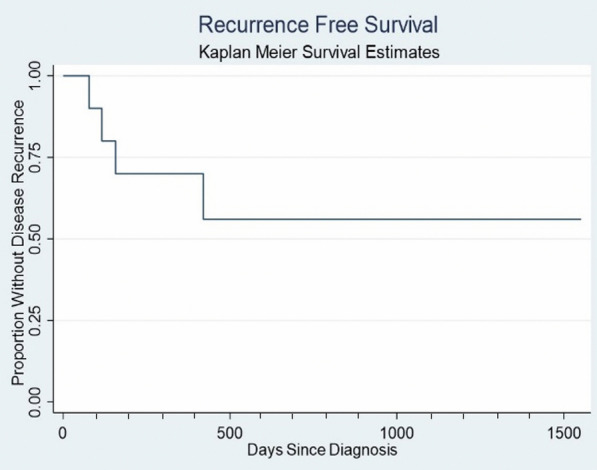
Kaplan Meier survival analysis depicts the time to disease recurrence after last intravitreal methotrexate injection. Note: Minor tick marks denote 100-day intervals

Mean time ± SD to either PR or CR was 57 ± 37 days, and 6 eyes (60%) exhibited at least partial regression with no relapse after local treatment. At final follow-up, 8 eyes showed complete regression (80%) with two eyes demonstrating stable PR (20%) although there were relapses that required repeat treatment. For the 4 eyes that experienced a relapse during the follow-up period, average time to first relapse was 6.5 ± 5.2 months, and one eye experienced a second relapse. Kaplan–Meier analysis of recurrence-free survival showed that most recurrences occurred within two months of the last MTX injection (Fig. 1). Three out of 4 patients with subretinal lesions showed complete regression at final follow-up with fewer than 4 doses of intravitreal treatment. Notably, three of the 4 eyes with vitreous disease showed a CR after fewer than 4 doses of intravitreal MTX.

When evaluating visual acuity on a per eye basis, visual acuity showed improvement in 4 eyes (40%), no change in one eye (10%), and decreased in five eyes (50%). The causes for visual decline in the five eyes over long-term follow-up included the following: Relapse of lymphoma within macula (1 eye), progression of macular degeneration (2 eyes, one wet and one dry macular degeneration), cataract progression where patient deferred surgery (1 eye), and undetermined (1).

**Fig. 2:**
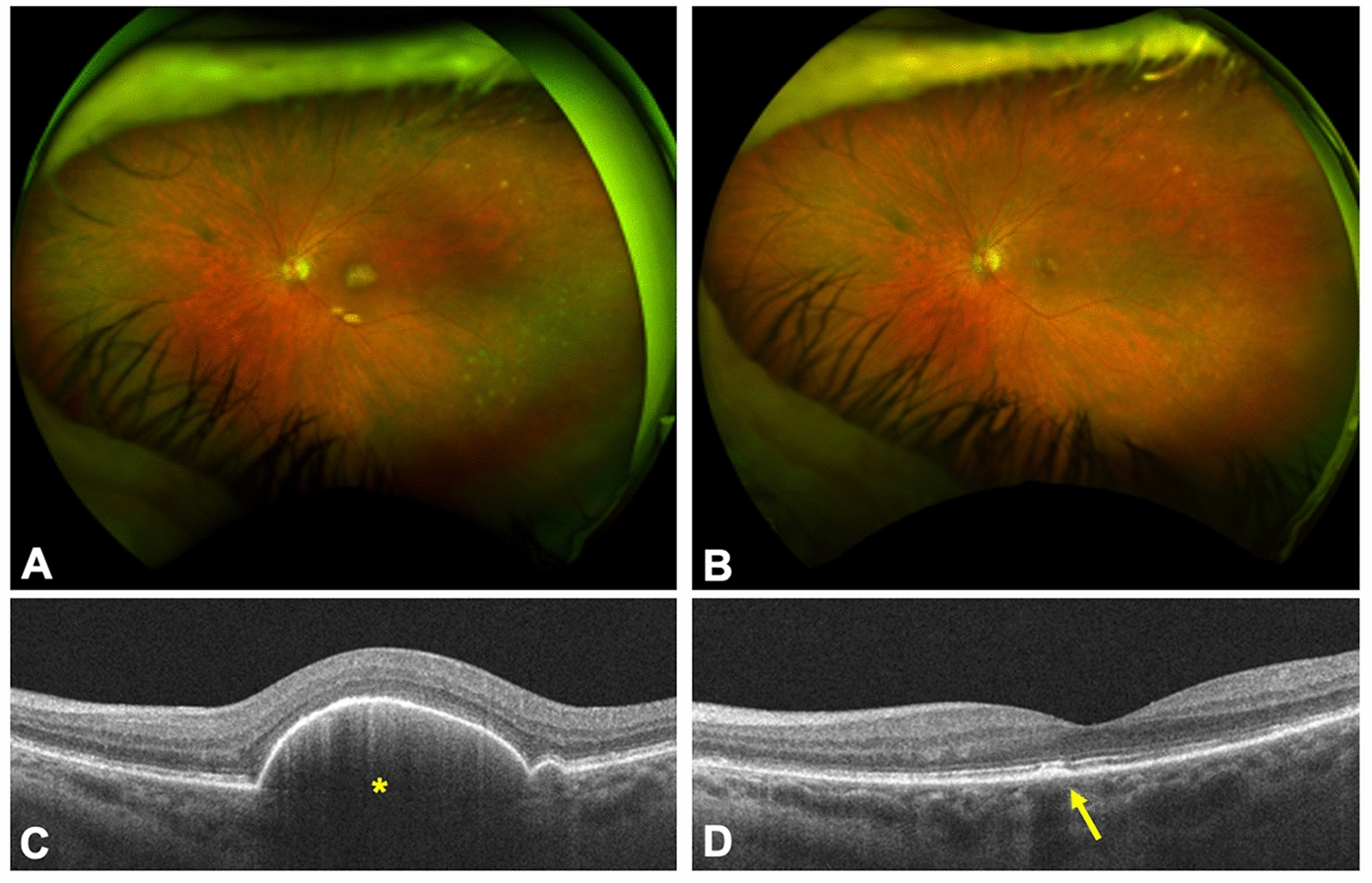
**A** Wide-field fundus photos of a patient with a history of central nervus system lymphoma and primary vitreoretinal lymphoma involving the subretinal and subretinal pigment epithelium tissue layers with **B** fundus photo demonstrating disease regression one month after intravitreal methotrexate. **C** Optical coherence tomography scan shows lesion under the retinal pigment epithelium prior to intravitreal methotrexate (asterisk). **D** Mild outer retinal irregularities and RPE changes are observed following treatment

Figure 2 shows the fundus photographs of a 66-year-old female (Patient 4) with a history of cerebellar DLBCL who presented with a fovea-involved, cream-colored subretinal lesion OS. She received one injection of MTX with complete regression of the subretinal lesion after one month with only mild residual outer retinal irregularities and improved visual acuity from 20/60 to 20/40. She remained stable with a CR at final 14-month follow-up.

**Fig. 3:**
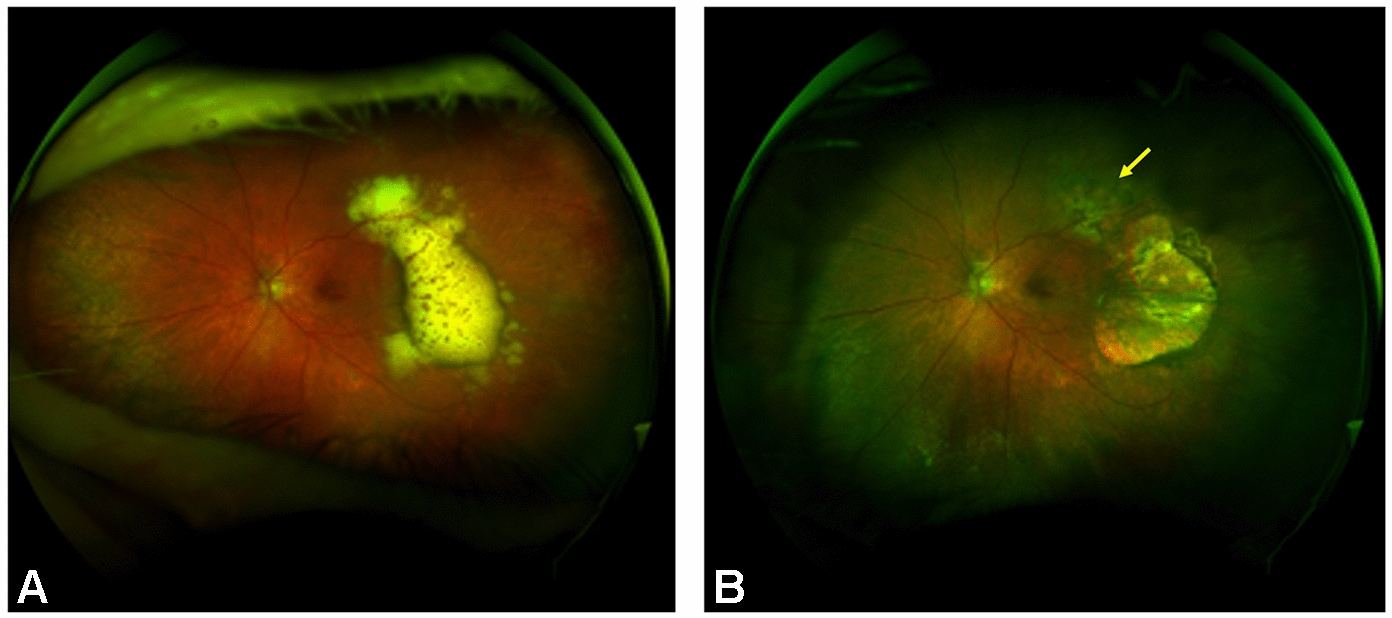
**A** Wide-field fundus photo shows a multilobulated crescentic subretinal lesion temporal to the macula, which was diagnosed primary vitreoretinal lymphoma following pars plana vitrectomy with subretinal biopsy. **B** Following two intravitreal methotrexate injections, a well-circumscribed atrophic area is observed with a few satellite lesions (arrow) superior to the macula at 3-month follow-up

Figure 3 shows an 86-year-old female (Patient 6) with no known history of CNS lymphoma who developed a temporal crescentic subretinal lesion OS and multifocal satellite lesions superior to the crescent-shaped lobulated lesion. The patient underwent pars plana vitrectomy and biopsy of the subretinal lesion, which confirmed lymphoma. She subsequently received two injections of intravitreal MTX with no evidence of active disease after 3 months. The larger lesion and satellite lesions completely regressed with no evidence of disease recurrence or non-ocular CNS involvement with MRI surveillance at final 49-month follow-up.

However, one of the two patients with PVRL who had no prior history of CNS lymphoma (Patient 1) developed non-ocular CNS lymphoma 32 months after her initial diagnosis of PVRL, a reminder of the variability of disease course and need to continue follow-up of both ocular and CNS disease surveillance. Of the 51 MTX injections administered over, no occurrences of retinal detachment, endophthalmitis, corneal epitheliopathy or serious adverse events were observed.

## Discussion

In this retrospective cohort of PVRL patients treated with intravitreal MTX, local therapy administered on a monthly basis was well-tolerated and led to a positive response in the majority of patients at approximately 2 months after initiation of treatment and 80% of patients showed a complete response at final follow-up. While our sample size was limited, patients experienced PR or CR without progressive ocular disease whether they presented with subretinal or vitreous involvement. Moreover, their visual acuities remained stable during the 12-month period following intravitreal MTX although a non-statistically significant improvement was observed with a mean follow-up of over 2 years. While many clinicians utilize systemic MTX to prevent progression of PVRL to non-ocular disease, only one patient in this series treated with local MTX developed non-ocular CNS lymphoma after PVRL diagnosis.

The treatment of PVRL varies in the literature with treatment modalities that may include systemic therapy, often with high-dose intravenous MTX, intrathecal chemotherapy for individuals with meningeal involvement and local therapies that include intravitreal MTX, intravitreal rituximab, or radiation [5–11]. Combination systemic and intravitreal strategies have also been employed but can be limited by systemic side effects. Local adverse effects associated with intravitreal MTX may include corneal epitheliopathy, dry eyes, and retinal pigment epithelial (RPE) abnormalities [8]. However, whether MTX contributes to RPE abnormalities or whether these findings are due to underlying PVRL is unclear.

Prior dosing strategies have included twice weekly MTX injections as induction therapy for 1 month followed by weekly MTX injections until disease regression. However, these protocols led to the development of corneal epitheliopathy 58% of cases [8]. However, no patients treated with a more frequent dosing regimen demonstrated irreversible vision loss attributable to methotrexate injections [8]. Combination intravitreal rituximab and MTX have also been employed, but the total volume of medication administered (0.2 cc) also requires management of post injection ocular hypertension, which may also include an anterior chamber paracentesis and associated risk [13, 14]. Given the potential adverse events associated with increased numbers of methotrexate injections, our experience with intravitreal MTX dosed monthly showed promising disease regression with a favorable safety profile. In addition, our management using a multi-disciplinary approach with a Neuro-Oncology service, predominantly employing local treatment in the setting of a normal MRI and no clear neurologic involvement. It is also notable that five of 7 patients had a prior history of systemic chemotherapy and decision-making was also made with scheduled surveillance by the Neuro-Oncology team with both non-ocular CNS and systemic surveillance with radiographic imaging. In this context, focal intravitreal treatment may offer benefits in avoiding potential toxicities associated with high-dose chemotherapy. This more conservative protocol showed complete response in the majority of patients at final follow-up, while 1 patient developed CNS involvement during the follow-up period.

Limitations of this study include the small number of patients and variability of follow-up. Our small sample size may impact the generalizability of the data. Specifically, other series have demonstrated a longer duration prior to disease relapse of up to 19 months [9] compared to 6.5 months observed in our series The few relapses in our series (n = 4 eyes) are thus difficult to compare to larger, multicenter data sets and further understanding of risk factors for PVRL relapse will be helpful in the future for patient monitoring. Moreover, the retrospective nature of this report limits our analysis of other variables that may be related to disease relapse or response, including intravitreal injection interval and standardization of systemic chemotherapy when administered.

Nonetheless, in this small, uncontrolled retrospective series, PR and CR was observed at approximately 2 months following initiation of therapy with few relapses and a well-tolerated toxicity profile. Further studies on the efficacy of intravitreal treatment alone in select patients versus combined systemic and intravitreal treatment are warranted to determine the efficacy, safety, and risk of ocular or non-ocular CNS relapse with intravitreal therapy.

## Data Availability

Data are presented in Table 1 and may be made available when requested.
